# Supplementary material for: New Benthic Cyanobacteria from Guadeloupe Mangroves as Producers of Antimicrobials
Source: Mar Drugs. 2019 Dec 23;18(1):16. doi: 10.3390/md18010016 (PMC7024286; doi:10.3390/md18010016)
Supplement: Supplementary file 1 [file marinedrugs-18-00016-s001.zip › Figure S2.pdf]

Anacyclamide A10
PMC 1092.19 (*Lyngbya* sp.)

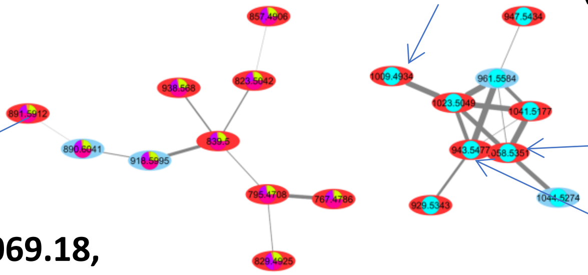

Wewakazole
PMC 1092.19
(*Lyngbya* sp.)

Wewakamide A
PMC 1057.18, PMC 1069.18,
PMC 1050.18, PMC 1052.18
(*Arthrospira* sp., *Oscillatoria* sp.,
Scytonema sp., *New genus* 3)

Nostophycin
PMC 1092.19 (*Lyngbya* sp.)

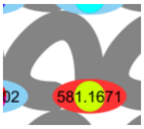

Raocyclamide B
PMC 1056.18, PMC 1057.18
(*Arthrospira* sp.)

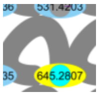

Aerucyclamide C
PMC 1092.19 (*Lyngbya* sp.)

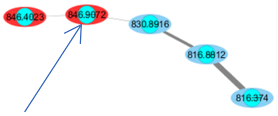

Microcystin LR
PMC 1092.19 (*Lyngbya* sp.)

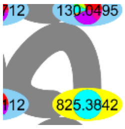

Microcystin RA
PMC 1092.19
(*Lyngbya* sp.)

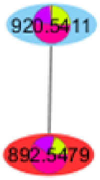

Viequeamide A
PMC 1050.18, PMC 1052.18,
PMC 1069.18, PMC 1057.18
(*Oscillatoria* sp., *New genus* 3,
Scytonema sp., *Arthrospira* sp.)

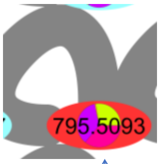

Pitipeptolide A
PMC 1050.18, PMC 1069.18, PMC 1057.18
(*Oscillatoria* sp., *Scytonema* sp., *Arthrospira* sp.)

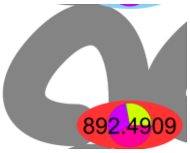

Majusculamide C Demethoxy
PMC 1050.18, PMC 1069.18, PMC 1057.18
PMC 1075.18
(*Oscillatoria* sp., *Scytonema* sp., *Arthrospira* sp.)
