# Supplementary material for: New Benthic Cyanobacteria from Guadeloupe Mangroves as Producers of Antimicrobials
Source: Mar Drugs. 2019 Dec 23;18(1):16. doi: 10.3390/md18010016 (PMC7024286; doi:10.3390/md18010016)
Supplement: Supplementary file 1 [file marinedrugs-18-00016-s001.zip › marinedrugs-666775_Supplementary Materials.pdf]

Supplementary Materials: New Benthic Cyanobacteria from Guadeloupe Mangroves as Producers of Antimicrobials

Sébastien Duperron,[1,2,*], Mehdi A. Beniddir [3], Sylvain Durand [1], Arlette Longeon [1], Charlotte Duval [1], Olivier Gros [4], Cécile Bernard [1] and Marie-Lise Bourguet-Kondracki [1,*]

[1] Molécules de Communication et Adaptation des Microorganismes, UMR 7245 CNRS, Muséum National d'Histoire Naturelle, 57 rue Cuvier (CP54), 75005 Paris, France; sylvain.durand@orange.fr (S.D.); arlette.longeon@mnhn.fr (A.L.); charlotte.duval@mnhn.fr (C.D.); cecile.bernard@mnhn.fr (C.B.)

[2] Institut Universitaire de France, 75005 Paris, France.

[3] Équipe "Pharmacognosie-Chimie des Substances Naturelles" BioCIS, CNRS, Université Paris-Saclay 5 rue Jean-Baptiste Clément, 92290 Châtenay-Malabry, France; mehdi.beniddir@u-psud.fr

[4] UMR 7205 ISYEB et Université des Antilles, Pointe à Pitre, 97157 Guadeloupe, France; olivier.gros@univ-antilles.fr

* Correspondence: sebastien.duperron@mnhn.fr (S.D.); marie-lise.bourguet@mnhn.fr (M.-L.B.-K)

Table S1: Pairwise distance values among new cyanobacterial strains isolated during this study. Values below 0.05 (5% divergence, as congenerics) are in bold.

Table S2. List of the 54 candidate structures, which are consistent with previously identified peptides using DEREPLICATOR algorithm.

Figure S1. Global molecular network obtained from LC-MS/MS data of 20 cyanobacteria extracts (red ellipses are DEREPLICATOR peptide matches).

Figure S2. A selection of clusters and self-loops annotated with putative cyanobacterial peptides and their origin.
