# Supplementary material for: New Benthic Cyanobacteria from Guadeloupe Mangroves as Producers of Antimicrobials
Source: Mar Drugs. 2019 Dec 23;18(1):16. doi: 10.3390/md18010016 (PMC7024286; doi:10.3390/md18010016)
Supplement: Supplementary file 1 [file marinedrugs-18-00016-s001.zip › Table S2.pdf]

Table S2. List of 54 candidate structures, which are consistent with previously identified peptides using DEREPLICATOR algorithm.

Compound	Producer	Phylum	Activity	Ref	Found in strain PMC*	Score	P-value	PepMass	SpecMass
Stylissamide C	*Stylissa caribica*	Porifera		1	1069, 1070, 1050, 1052, 1057	14	6.5 10^{-20}	861.44	829.49
Sanguinamide_A	*Hexabranchus sanguineus*	Mollusca (Nudibranch)		2	1069, 1070, 1050, 1052, 1057	13	1.0 10^{-17}	721.36	767.48
E'Champacyclin'	*Streptomyces* C42	Bacteria	Antibacterial	3	1069, 1070, 1050, 1052, 1057	13	4.4 10^{-17}	897.61	910.57
B_12489_B_12489C	Unknown			-	1092	15	5.7 10^{-18}	1114.64	989.57
Gramicidin S2	*Aneurinibacillus migulanus*	Bacteria	Antibacterial	4	1050, 1074, 1066, 1080, 1056, 1069, 1057	13	1.1 10^{-15}	1126.69	1098.63
Viequeamide A	*Rivularia* sp	Cyanobacteria	Cytotoxic	5	1050, 1052, 1069,	12	2.8 10^{-17}	803.50	892.55

					1057				
Dominicin	*Eurypon laughlini*	Porifera		6	1052, 1050, 1069, 1057	12	$2.1\ 10^{-15}$	844.54	901.57
Wewakamide A	*Lyngbya semiplena* *Lyngbya majuscula*	Cyanobacteria	Cytotoxic	7	1057, 1069, 1050, 1052,	12	$4.4\ 10^{-15}$	994.65	891.59
Wewakazole	*Lyngbya majuscula*	Cyanobacteria		8	1092, 1056	12	$6.7\ 10^{-12}$	1140.54	1058.54
Nostophycin	*Nostoc* 152	Cyanobacteria	Cytotoxic	9	1092	11	$2.1\ 10^{-15}$	888.48	943.55
Phakellistatin 2	*Phakellia carteri*	Porifera	Cytotoxic	10	1052, 1050, 1064, 1056, 1069, 1057, 1068	11	$1.2\ 10^{-14}$	827.46	811.50
Rolloamide A	*Eurypon laughlini*	Porifera	Cytotoxic	11	1057, 1069, 1056, 1080, 1064, 1051, 1050	11	$1.5\ 10^{-14}$	763.46	795.47
Majusculamide C_Demethoxy	*Lyngbya majuscula*	Cyanobacteria	Cytotoxic	12	1057, 1069, 1050, 1075	11	$4.6\ 10^{-13}$	954.58	892.49
Callyaerin H	*Callyspongia aerizusa*	Porifera	Cytotoxic	13	1070, 1052, 1050, 1069, 1057	10	$1.8\ 10^{-13}$	1043.62	938.57
Hymenamide B	*Hymeniacidon*	Porifera		14	1092	10	$4.0\ 10^{-13}$	830.40	830.39

Haliclonamide B	Marine sponges	Porifera		15	1092	10	5.4 10^{-12}	788.39	846.40
Empedopeptin	*Empedobacter haloabium nov*. sp. (ATCC 31962).	Bacteria	Antibacterial	16	1092	10	7.5 10^{-12}	1125.56	1051.51
Anacyclamide A10	*Anabaena* sp. *90*	Cyanobacteria	Cytotoxic	17	1092	10	9.6 10^{-11}	1052.53	1009.49
Micropeptin_SD944	*Microcystis aeruginosa*	Cyanobacteria		18-	1092	9	1.4 10^{-14}	944.52	929.53
Destruxins 2-Valine analogue5'-hydroxy,	*Beauveria felina*	Fungus	Insecticid Cytotoxic	19	1057	9	1.6 10^{-11}	595.36	593.29
Cycloamanide_D	*Amanita phalloides*	Fungus	Immunosuppressive	20	1052, 1050, 1069, 1057,	9	1.5 10^{-10}	884.48	857.49
Myriastramide_A	*Myriastra clavosa*	Porifera		21	1052, 1050, 1069, 1057	9	1.6 10^{-10}	854.43	839.50
Myriastramide_A_?3''-Isomer,_2''?-chloro	Unknown			-	1092	9	1.6 10^{-10}	888.39	846.38
Aureobasidin N	*Aureobasidium pullulans*	Marine-Derived Fungus	Antifungal	22	1092	9	4.2 10^{-10}	1082.68	975.55
Sanguinamide B	*Hexabranchus sanguineus*	Mollusca (Nudibranch)		2	1092	9	1.1 10^{-9}	710.27	677.22
Deoxy Mozamide B	*Theonella* sp.	Porifera		23	1071	8	6.7 10^{-12}	844.49	832.52
Stylissamide E	*Stylissa caribica*	Porifera		24	1092	8	6.5 10^{-11}	782.43	681.30
Ceratospongamide	Red alga *Ceratodictyon spongiosum &* symbiotic sponge *Sigmadocia.*	Algae and Porifera		25	1069, 1057, 1050	8	8.3 10^{-11}	767.35	799.44
Xentrivalpeptide P	*Xenorhabdus* sp.	Bacteria		26	1050,	8	9.8 10^{-11}	797.47	782.48

					1069, 1057				
Lissoclinamide 1	*Lissoclinum patella*	Chordata (Ascidian)	Cytotoxic	27	1076, 1071, 1052, 1050, 1069, 1057, 1068	8	$4.9\ 10^{-10}$	705.28	659.39
Kulokekahilide 1	*Cephalaspidean mollusc Philinopsis speciosa*	Mollusca	Cytotoxic	28	1092	8	$7.1\ 10^{-10}$	954.55	1069.53
Iturin A_Iturin A3	*Bacillus subtilis*	Bacteria	Antibacterial	29	1092	8	$8.7\ 10^{-10}$	1056.56	1041.52
Iturin A_Iturin A2	*Bacillus subtilis*	Bacteria	Antibacterial	29	1092	8	$1.0\ 10^{-9}$	1042.54	1023.50
Haliclonamide_B_O-(2-Hydroxy-3-methyl-3-butenyl)	*Haliclona* sp	Porifera		30-	1092	8	$1.6\ 10^{-9}$	872.44	947.54
Citrusin VIII	*Citrus medica*	Plant, fruit peel		31	1070, 1069	8	$5.9\ 10^{-9}$	821.48	782.41
Pteratide III	*Basidiomycete Pterula*	Fungus	Cytotoxic	32	1070, 1052, 1050, 1074, 1080, 1069, 1057, 1072	7	$5.9\ 10^{-10}$	818.49	812.49
Vioprolide C	Myxobacterium *Cystobacter violaceus*	Bacteria	Antifungal Antibacterial	33	1071, 1052	7	$4.3\ 10^{-9}$	848.41	896.57
Etamycin_A_2-(N-Methyl-L-leucine)_analogue	*Streptomyces* sp.	Bacteria	Antibacterial	34	1057, 1069, 1050, 1052	7	$4.6\ 10^{-9}$	858.49	806.50
Xentrivalpeptide B	*Xenorhabdus* sp.	Bacteria		26	1050,	7	$4.7\ 10^{-9}$	783.45	810.47

					1069, 1057				
Daitocidin B2	*Bacillus pumilus*	Bacteria	Antibacterial	35-	1050, 1069, 1057	7	5.9 10^{-9}	895.53	893.53
Dolastatin I	*Dolabella auricularia*	Mollusca *(Sea hare)*	Cytotoxic	36	1092	7	6.3 10^{-9}	516.22	577.24
Tunicyclin D	*Psammosilene tunicoides*	Plant	Antifungal	37	1052, 1050, 1069, 1057	7	7.0 10^{-9}	900.46	823.50
Hymenamide F	*Hymeniacidon* sp.	Porifera		38	1057, 1069, 1050, 1052, 1070	7	8.8 10^{-9}	764.44	768.47
Aerucyclamide C	*Microcystis aeruginosa*	Cyanobacteria		39	1092	7	9.5 10^{-9}	516.22	645.28
[D-Asp3,(E)-Dhb7]Microcystin LR_[D-Asp3,(E)-Dhb7]Microcystin LR	*Microcystis bloom*	Cyanobacteria	Cytotoxic	40	1092	7	1.5 10^{-8}	980.53	846.91
Pitipeptolide A	*Lyngbya majuscula*	Cyanobacteria		41	1057, 1069, 1050	7	1.6 10^{-8}	807.48	795.51
Microcystin RA	*Microcystis*	Cyanobacteria		40	1092	7	1.7 10^{-8}	952.50	825.38
Prepatellamide A	*Lissoclinum patella*	Chordata, (Ascidian)		42	1052, 1056, 1064, 1051, 1050	7	2.7 10^{-8}	760.34	657.29
Cyclonellin	*Axinella carteri*	Porifera		43	1080, 1069, 1072, 1074,	6	1.8 10^{-8}	962.46	960.59

					1051, 1070, 1092				
Lissoclinamide 9	*Lissoclinum patella*	Ascidian	Cytotox	27	1056, 1057	6	$2.1\ 10^{-8}$	707.29	593.29
Kailuin D	A marine-derived bacterium	Marine bacteria	Cytotox	44	1071,	6	$4.9\ 10^{-8}$	751.51	804.52
Raocyclamide B	*Oscillatoria raoi*	Cyanobacteria		45	1056, 1057	6	$6.5\ 10^{-8}$	568.21	581.17
Viomycin,_BAN,_INN_4'-Hydroxy	*Streptomyces*	Bacteria	Antibacterial, Antituberculosis	46	1050, 1051	6	$6.8\ 10^{-8}$	701.32	602.26
Sclerotide A	*Aspergillus sclerotiorum PT06-1*	Marine-Derived Fungus	Antifungal	47	1052,	6	$7.3\ 10^{-8}$	709.29	705.31

* : Code strain : for all strains, read PMC XXXX.18 except for PMC 1092.19

References

1. Schmidt, G.; Grube, A. Stylissamides A-D, new proline-containing cyclic fleptapeptides from the marine sponge *Stylissa caribica. Eur. J. Org. Chem.* **2007**, 4103-4110.

2**.** Dalisay, D.S.; Rogers, E.W.; Edison, A.S.; Molinski, T.F. Structure Elucidation at the Nanomole Scale. 1. Trisoxazole Macrolides and Thiazole-Containing Cyclic Peptides from the Nudibranch *Hexabranchus sanguineus. J. Nat. Prod*. **2009**, *72*, 732-738.

3. Pesic, A.; Baumann, H.I.; Kleinschmidt, K.; Ensle, P.; Wiese, J. Champacyclin, a New Cyclic Octapeptide from *Streptomyces* Strain C42 Isolated from the Baltic Sea. *Mar. Drugs* **2013**, *11*, 4834-4857.

4. Wenzel, M.; Rautenbach, M.; Vosloo, J.A.; Siersma, T.; Aisenbrey, C.H.M.; Ek.Zaitseva, E.; Laubscher, W.E.; van Rensburg, W.; Behrends, J.C.; Bechinger, B.; Hamoen L.W. The Multifaceted Antibacterial Mechanisms of the Pioneering Peptide Antibiotics Tyrocidine and Gramicidin S. *mBio*. **2018**, 9(5) 802-818.

5. Boudreau, P.D.; Byrum, T.; Liu, W.T.; Dorrestein, P.C.; Gerwick, W.H. Viequeamide A, a Cytotoxic Member of the Kulolide Superfamily of Cyclic Depsipeptides from a Marine Button Cyanobacterium. *J. Nat. Prod.* **2012**, *75*, 1560-1570.

6. Williams, D.E.; Patrick, B O.; Behrisch, H.W. Dominicin, a cyclic octapeptide, and laughine, a bromopyrrole alkaloid, isolated from the Caribbean marine sponge *Eurypon laughlini. J. Nat. Prod.* **2005**, *68*, 327-330.

7. Han, B.N.; Gross, H.; McPhail, K.L.; Goeger, D.; Maier, C.S.; Gerwick, W.H. Wewakamide A and Guineamide G, Cyclic Depsipeptides from the Marine Cyanobacteria *Lyngbya semiplena* and *Lyngbya majuscula. J. Microbiol. Biotechnol.* **2011**, *21*, 930-936.

8. Nogle, L.M.; Marquez, B.L.; Gerwick, W.H. Wewakazole, a novel cyclic dodecapeptide from a Papua New Guinea *Lyngbya majuscula*. *Org. Lett.* **2003**, *5*, 3-6.

9. Fujii, K.; Sivonen, K.; Kashiwagi, T.; Hirayama, K.; Harada, K. Nostophycin, a Novel Cyclic Peptide from the Toxic Cyanobacterium *Nostoc* sp. 152. *J. Org. Chem.* **1999**, *64*, 16, 5777-5782.
10. Pettit, G.R.; Tan, R.; Williams, M.D.; Tackett, L.; Schmidt, J.M.; Cerny, R L.; Hooper, J.N.A. Isolation and structure of phakellistatin 2 from the Eastern Indian Ocean marine sponge *Phakellia carteri*. *Bioorg. Med. Chem. Lett.* **1993**, *3*, 2869-2874.
11. Williams, D.E.; Yu, K.; Behrisch, H.W. Rolloamides A and B, Cytotoxic Cyclic Heptapeptides Isolated from the Caribbean Marine Sponge *Eurypon laughlini. J. Nat. Prod.* **2009**, *72*, 1253-1257.
12. Carter, D.C.; Moore, R.E.; Mynderse, J.S.; Niemczura, W.P.; Todd J.S. Structure of Majusculamide C ; a cyclic depsipeptide from *Lyngbya majuscula*. *J. Org. Chem.* **1984**, *49*, 236-241.
13. Ibrahim, S.R.M.; Min, C.C.; Teuscher, F.; Ebel, R.; Kakoschke, C.; Lin, W.H.; Wray, V.; Edrada-Ebel, R.; Proksch, P. Callyaerins A-F and H, new cytotoxic cyclic peptides from the Indonesian marine sponge *Callyspongia aerizusa. Bioorg. Med. Chem.* **2010**, *18*, 4947-4956.
14. Kobayashi, J.; Tsuda, M.; Nakamura, T.; Mikami, Y.; Shigemori, H. Hymenamides A and B, new proline-rich cyclic heptapeptides from the Okinawan marine sponge *Hymeniacidon* sp. *Tetrahedron* **1993**, *49*, 2391-2402.
15. Guan, L. L.; Sera, Y.; Adachi, K.; Nishida, F.; Shizuri, Y. Isolation and evaluation of nonsiderophore cyclic peptides from marine sponges. *Biochem. Biophys. Res. Commun.* **2001**, *283*, 976-981.
16. Konishi, M.; Sugawara, K.; Hanada, M.; Tomita, K.; Tomatsu, K.; Miyaki, T.; Kawaguchi, H.; Buck, R.E.; More, C.; Rossomano, V.Z. Empedopeptin (BMY-28117), a new depsipeptide antibiotic. I. Production, isolation and properties. *J Antibiot (Tokyo)* **1984**, *37*(9), 949-957.
17. Fewer, D.P.; Jokela, J.; Wahlsten, M.; Rouhiainen, L.; Sivonen, K. Highly Diverse Cyanobactins in Strains of the Genus *Anabaena. Appl. Environ. Microbiol.* **2010**, *76*(3), 701–709.
18. Yamaki, H.; Sitachitta, N.; Sano, T.; Kaya, K. Two New Chymotrypsin Inhibitors Isolated from the Cyanobacterium *Microcystis aeruginosa* NIES-88. *J. Nat. Prod.* **2005**, *68*, 1, 14-18.
19. Lira, S.P.; Vita-Marques, A.M.; Seleghim, M.H.R.; Bugni, T S.; LaBarbera, D V.; Sette, L.D.; Sponchiado, S.R.P. New destruxins from the marine-derived fungus *Beauveria felina*. *J. Antibiot.* **2006**, *59*, 553-563.
20. Wieczorek, Z.; Siemion, I.Z.; Zimecki, M.; Bolewska-Pedyczak, E.; Wieland, T. Immunosuppressive Activity in the Series of Cycloamanide Peptides from Mushrooms. *Peptides* **1993**, *14*, 1-5.
21. Erickson, K.L.; Gustafson, K.R.; Milanowski, D.J.; Pannell, L.K.; Klose, J.R.; Boyd, M.R. Myriastramides A-C, new modified cyclic peptides from the Philippines marine sponge *Myriastra clavosa. Tetrahedron* **2003**, *59*, 10231-10238.
22. Takesako, K.; Ikai, K.; Haruna, F.; Endo, M.; Shimanaka, K.; Sono, E.; Nakamura, T.; Kato, I.; Yamaguchi, H. Aureobasidins, new antifungal antibiotics. Taxonomy, fermentation, isolation, and properties. *J Antibiot* **1991,** *44*(9), 919-924.21.
23. Schmidt, E.W.; Harper, M.K.; Faulkner, D.J. Mozamides A and B, Cyclic Peptides from a *Theonellid* Sponge from Mozambique. *J. Nat. Prod.* **1997**, *60*, 8, 779-782.
24. Cychon, C.; Kock, M. Stylissamides E and F, Cyclic Heptapeptides from the Caribbean Sponge *Stylissa caribica*. *J. Nat. Prod.* **2010**, *73*, 738-742.
25. Tan, L.T.; Williamson, R.T.; Gerwick, W.H.; Watts, K.S.; McGough, K.; Jacob,s R. Cis,cis- & trans,trans-ceratospongamide, new bioactive cyclic heptapeptides from the Indonesian red alga *Ceratodictyon spongiosum* & symbiotic sponge *Sigmadocia*. *J. Org. Chem.* **2000**, *65*, 419-425.

26. Zhou, Q.; Dowling, A.; Heide, H.; Wöhnert, J.; Brandt, U.; Baum, J.; Constant, R.; Bode, H.B. Xentrivalpeptides A–Q: Depsipeptide Diversification in *Xenorhabdus. J. Nat. Prod.* **2012**, *75*, 10, 1717-1722.
27. Morris, L.A. ; Van den Bosch, J.J.K.; Versluis, K.; Thompson, G.S.; Jaspars, M. Structure determination and MSn analysis of two new lissoclinamides isolated from the Indo-Pacific ascidian *Lissoclinum patella*. *Tetrahedron* **2000**, *56*, 42, 8345-8353.
28. Kimura J.; Takada, Y.; Inayoshi, T.; Nakao, Y.; Goetz, G.; Yoshida, W.Y.; Scheuer, P.J. Kulokekahilide-1, a cytotoxic depsipeptide from the cephalaspidean mollusc *Philinopsis speciosa. J. Org. Chem.* **2002**, *67*, 1760-1767.
29. Noriyasu, I.; Shahedur, R.M.; Takashi, A. Production of iturin A homologues under different culture conditions. *Journal of Environmental Sciences* Supplement **2009,** S28–S32.
30. Guan, L.L.; Sera, Y.; Kadachi, K.; Nishida, F.; Shizuri, Y. Isolation and Evaluation of Nonsiderophore Cyclic Peptides from Marine Sponges. *Biochemical and Biophysical Research Communications*, **2001,** 283 (4), 976-981.
31. Morita, H.; Takeya, K. Bioactive Cyclic Peptides from Higher Plants. *Heterocycles, Review* **2010**, *80* No. 2, 739-764.
32. Chen, C.H.; Lang, G.; Mitova, M.I.; Murphy, A.C.; Cole, A.L.; Din, L.B.; Blunt, J.W.; Munro, M.H. Pteratides I-IV, new cytotoxic cyclodepsipeptides from the Malaysian *basidiomycete Pterula* sp. *J Org Chem.* **2006**, *71*(21), 7947-7951.
33. Herrmann, J.; Abou Fayad, A.; Müller, R. Natural products from myxobacteria: novel metabolites and bioactivities. *Nat. Prod. Rep.* **2017**, *34*, 135-160.
34. Sun, P.; Maloney, K.N.; Nam, S.J.; Haste, N.M.; Raju, R.; Aalbersberg, W.; Jensen, P.R.; Nizet, V.; Hensler, M.E.; Fenical, W. Fijimycins A-C, three antibacterial etamycin-class depsipeptides from a marine-derived *Streptomyces* sp. *Bioorg. Med. Chem*. **2011**, *19*, 6557-6562.
35. Atta-ur-Rahman *Studies in Natural Products Chemistry*, Elsevier Science, **2000**, p.89.
36. Sone, H.; Kigoshi, H.; Yamada, K. Isolation and stereostructure of dolastatin I, a cytotoxic cyclic hexapeptide from the Japanese sea hare *Dolabella auricularia*. *Tetrahedron* **1997**, *53*, 8149-8154.
37. Tian, J.; Shen, Y.; Yang, X.; Liang, S.; Shan, L.; Li, H.; Liu, R.; Zhang, W. Antifungal cyclic peptides from *Psammosilene tunicoides*. *J Nat Prod*. **2010**, *73*(12), 1987-1992.
38. Tsuda, M.; Shigemori, H.; Kobayashi, J. Hymenamides A - K, new cyclic peptides from a marine sponge *Hymeniacidon sp*. *Pept. Chem*. **1996**, *33*, 161-164.
39. Portmann, C.; Blom, J. F.; Kaiser, M.; Brun, R.; Juttner, F.; Gademann, K. Isolation of Aerucyclamides C and D and Structure Revision of Microcyclamide 7806A : Heterocyclic Ribosomal Peptides from *Microcystis aeruginosa* PCC 7806 and their Antiparasite Evaluation. *J. Nat. Prod*. **2008**, *71*, 1891–1896.
40. Matthiensen, A.; Beattie, K.A.; Yunes, J. S.; Kaya, K.; Codd, G.A. [D-Leu1]microcystin-LR, from the cyanobacterium *Microcystis* RST 9501 and from a *Microcystis* bloom in the Patos lagoon estuary, Brazil, *Phytochemistry* **2000**, *55*, 383-387.
41. Luesch, H.; Pangilinan, R.; Yoshida, W.Y.; Moore, R.E.; Paul, V.J. Pitipeptolides A and B, new cyclodepsipeptides from the marine cyanobacterium *Lyngbya majuscula*. *J. Nat. Prod.* **2001**, *64*, 304-307.
42. Fu, X.; Su, J.; Zeng, L. Prepatellamide A, a new cyclic peptide from the ascidian *Lissoclinum patella*. *Science in China Ser. B* **2000**, *43*, 643-648.
43. Milanowski, D.J.; Rashid, M.A.; Gustafson, K.R. Cyclonellin, a new cyclic octapeptide from the marine sponge *Axinella carteri. J. Nat. Prod.* **2004**, *67*, 441-444.
44. Harrigan, G.G.; Harrigan, B.L.; Davidson, B.S. Kailuins A-D, new cyclic acyldepsipeptides from cultures of a marine-derived bacterium. *Tetrahedron* **1997**, *53*, 1577-1582.

45. Admi, V.; Afek, U.; Carmeli, S. Raocyclamides A and B, novel cyclic hexapeptides isolated from the cyanobacterium *Oscillatoria raoi. J. Nat. Prod*. **1996**, *59*, 396-399.
46. Bycroft, B.W.; Payne, D.J. *Dictionary of Antibiotics and Related Substances*, **2013**, Page 1797.
47. Zheng, J.; Zhu, H.; Hong, K.; Wang, Y.; Liu, P.; Wang, X.; Peng, X.; Zhu, W. Novel Cyclic Hexapeptides from Marine-Derived Fungus, *Aspergillus sclerotiorum* PT06-1. *Org. Lett.* **2009**, *11*, 5262-5265.
