# Supplementary figures and images for: New Benthic Cyanobacteria from Guadeloupe Mangroves as Producers of Antimicrobials
Source: Mar Drugs. 2019 Dec 23;18(1):16. doi: 10.3390/md18010016 (PMC7024286; doi:10.3390/md18010016)

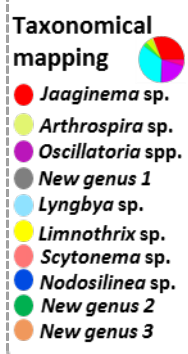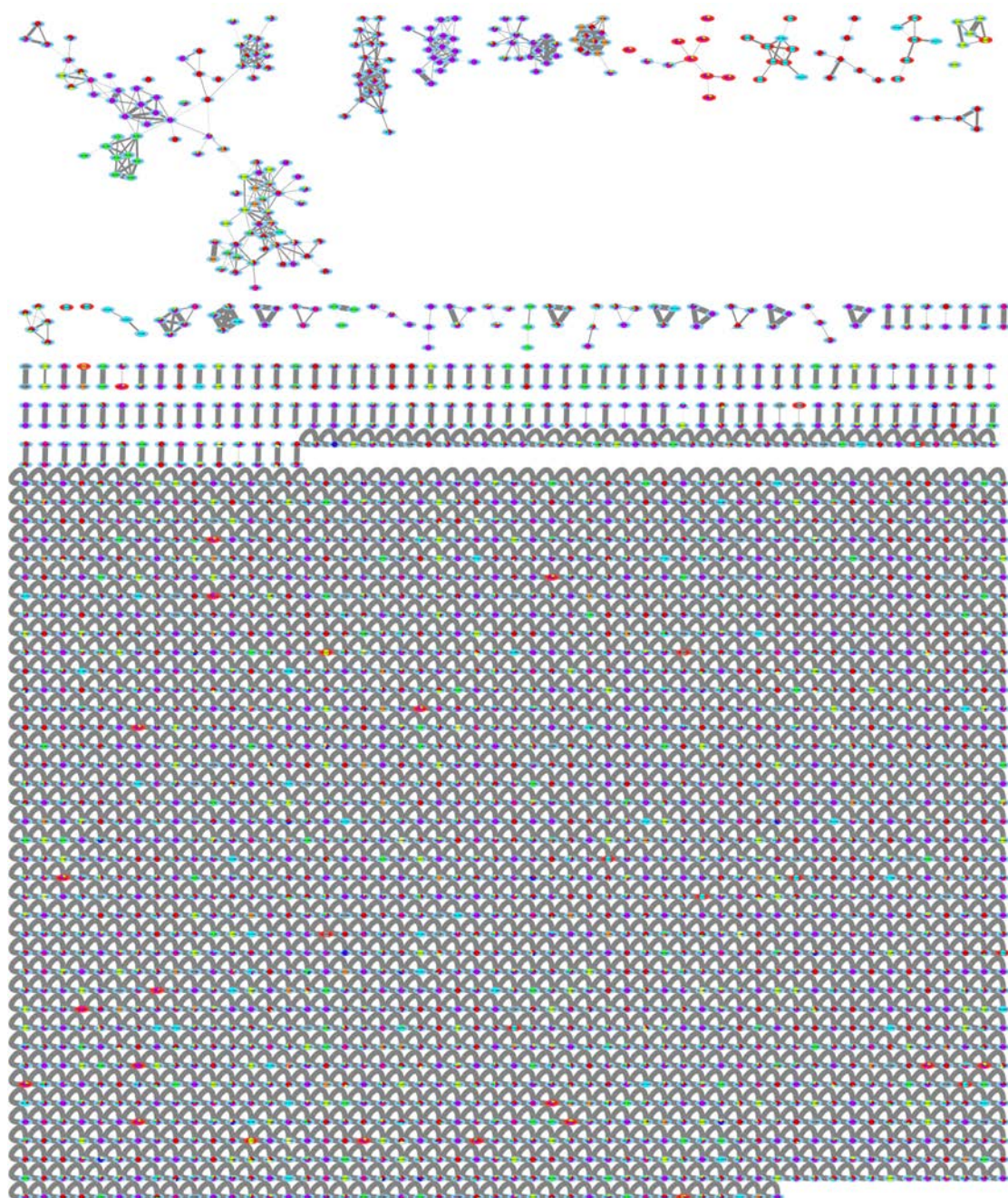

Supplement: Supplementary file 1 [file marinedrugs-18-00016-s001.zip › Figure S1.pdf]

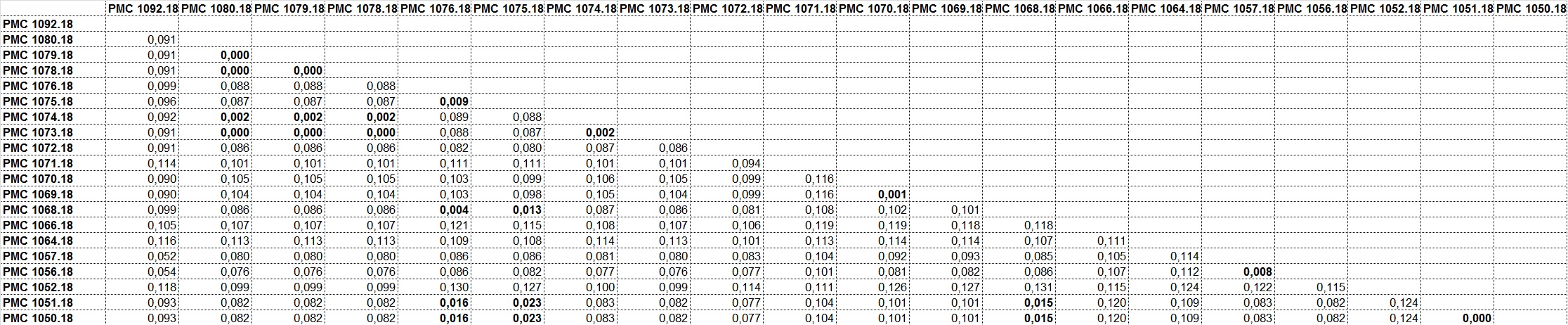

Supplement: Supplementary file 1 [file marinedrugs-18-00016-s001.zip › Table S1.jpg]
